# A Multicentre Epidemiologic Study of Sudden and Unexpected Death in Adult Cats and Dogs in Australia

**DOI:** 10.3390/vetsci10090582

**Published:** 2023-09-20

**Authors:** Mirrim Kelly-Bosma, Joerg Henning, Mark Haworth, Richard Ploeg, Lucy Woolford, Alison Neef, Shubhagata Das, Rachel Allavena

**Affiliations:** 1 School of Veterinary Science, The University of Queensland, Gatton, QLD 4343, Australiar.allavena@uq.edu.au (R.A.); 2 Faculty of Veterinary and Agricultural Sciences, University of Melbourne, Werribee, VIC 3030, Australia; 3 School of Animal and Veterinary Science, The University of Adelaide, Roseworthy, SA 5371, Australia; 4 School of Agricultural, Environmental and Veterinary Sciences, Charles Sturt University, Wagga Wagga, NSW 2650, Australia

**Keywords:** pathology, post-mortem, necropsy, sudden death, companion animal, cat, dog

## Abstract

**Simple Summary:**

Pets may die unexpectedly, without warning, and with no externally visible reason for death. As in people, postmortem examination can be performed to try to determine the cause of death. There are many diseases which may result in sudden unexpected death in domestic animals, although there is little research on companion animals and no studies performed in Australia. The aims of this study were to identify causes of sudden unexpected death in cats and dogs in Australia by examining postmortem reports and to identify risk factors for certain causes of sudden death.

**Abstract:**

Sudden and unexpected death (SUD) is a common reason for animals to undergo post-mortem examination. There is limited literature examining the causes of SUD in cats and dogs, and no research specific to Australia. The purpose of this study was to investigate the epidemiology and pathology of SUD in cats and dogs in a multicentric study across Australia. Retrospective post-mortem reports of SUD in cats and dogs were obtained from four veterinary schools in Australia distributed across four states. The frequency of SUD between institutes ranged from 2.1% to 6.5%. Dogs composed the majority of the study population (76%), and males outnumbered females, particularly in the feline subpopulation. After necropsy, 37% of SUD remained cause unknown, the largest category in both cats and dogs. When cause was identified, cardiovascular disease was most common in both species, followed by gastrointestinal disease in dogs, and trauma in cats. In dogs, multinomial logistic regression identified age as a risk factor significantly associated with the four largest categories of SUD. This study identified causes of SUD in Australian cats and dogs, including novel causes not previously reported. Further, this study revealed a higher rate of unsolved SUD in Australia than can be found in the literature from other countries.

## 1. Introduction

Post-mortem examination is a useful diagnostic tool in veterinary medicine, particularly recognized for its importance in production animal medicine and the maintenance of herd health. Additionally, it plays an important role in companion animal medicine, although it is often underutilized. It can fulfill many purposes including determining cause of death or illness, documenting disease progression and pathogenesis, investigating iatrogenic deaths and potential malpractice, and documenting animal cruelty cases. Small animal necropsies may be performed by veterinarians in clinical practice, or by veterinary pathologists in teaching institutions or commercial pathology laboratories.

Sudden and unexpected death (SUD) is a common reason for companion animals to present for post-mortem examination. Owners are often upset when a pet dies unexpectedly, and seek to understand why their pet passed away. SUD is recognized in human medicine, often as part of well-defined syndromes such as sudden infant death syndrome (SIDS) [1,2], sudden cardiac death in athletes [3], and sudden death in epilepsy [4].

The definition of ‘sudden’ death in human and veterinary medicine varies widely in the literature. In human medicine, one common definition of SUD is a natural fatal event that occurs within one hour of collapse [5,6]. Another defines SUD as immediate accidental death or death that occurs within 24 h from the onset of acute symptoms [7,8]. SUD is usually only applied to those patients with no prior history of illness. There is no clear definition in veterinary medicine, partially due to a paucity of literature. Parry (2008) describes SUD as death of an apparently healthy animal that occurs within minutes to hours of acute clinical signs [9]. Piegari et al. (2020) used the World Health Organization (WHO) definition of “non-violent and unexpected death that occurs less than 24 h after the onset of symptoms” [10]. A common definition in veterinary toxicology is death in animals previously perceived to be healthy with few or no proceeding observed clinical signs, or an extremely rapid time course. Defining ‘sudden’ death in animals can be problematic because animals may not be constantly observed. Therefore, death might appear to be sudden or unexpected to the observer, but the animal may have had unobserved or unrecognized clinical signs for a period prior to death.

SUD in people is investigated by autopsy, and can be classified according to cardiac and non-cardiac causes [7]. Cardiac causes are most common and include acute coronary syndrome, stress-induced myocardial ischemia, cardiomyopathy, congenital heart disease, arrhythmia, and viral myocarditis [6,7]. Non-cardiac causes are varied and include arrhythmogenic and cardiotoxic drugs, electrocution, acute respiratory disease (pulmonary embolism, pneumonia), acute gastrointestinal hemorrhage, epilepsy, anaphylaxis, necrotizing pancreatitis, acute peritonitis, and brain herniation [7,8].

There is limited literature exploring the causes of SUD in companion animals. Olsen and Allen (2000, 2001) described the causes of SUD in cats and dogs in Saskatchewan, Canada [11,12]. The authors defined SUD as animals that were found dead unexpectedly but were considered healthy when last observed by their owners [11,12]. In dogs, the most common cause of SUD was heart disease, followed by toxicity, gastrointestinal disease, and trauma [11]. In cats, trauma was the most common cause of SUD, followed by heart disease, intestinal disease, and respiratory disease [12]. The cause of death was undetermined in 12.6% of dogs and 12.7% of cats with SUD [11,12]. Another study focused on cardiac pathology as a cause of SUD in cats [13]. The authors reported a frequency of SUD of 12.9% over a 16-year study period in their study population, of which 55% were cardiac. The majority of cardiac SUD in cats was caused by hypertrophic cardiomyopathy (79.1%). Other causes included restrictive cardiomyopathy, endomyocarditis, and congenital abnormalities [13]. Piegari et al. (2020) investigated infectious causes of SUD in young dogs under 12 months old [10]. They reported a frequency of 14.5%, and those with SUD exhibited necropsy findings such as hemorrhagic gastroenteritis, pneumonia, and catarrhal enteritis. Microbiology was positive in 86% of cases, with parvovirus the most common etiologic agent isolated. Other etiologies included *Escherichia coli*, *Clostridium perfringens* type A, adenovirus, canine distemper virus, and *Pasteurella* spp. [10].

SUD has been more thoroughly investigated in horses, including the epidemiology and risk factors for race-associated death [14,15,16]. In studies of racing thoroughbred deaths on track or up to one hour after racing, causes included cardiac failure, respiratory failure, pulmonary haemorrhage, blood vessel rupture, vertebral fracture, spinal cord injury, and rupture of paralumbar musculature [17,18]. A definitive cause of death was identified in 53% of cases, a presumptive cause of death in 25%, and death remained unexplained in 22% of cases [17]. In a mixed population of horses and ponies, causes of SUD included cardiovascular disease, gastrointestinal disease, acute infections, trauma, and respiratory failure, and up to one third of SUD had no definitive diagnosis [19,20]. In cattle and sheep, literature on SUD is limited to editorials and instructive articles [21,22].

The current literature provides an indication of what disease processes can result in SUD in companion animals; however, the literature is sparse, is restricted to a few specific geographic locations, or is focused on a particular body system or process. While some causes of SUD are likely global, others may be region-specific. Australia contains many venomous and poisonous animals, and it is widely accepted by Australian veterinarians that fauna such as elapid snakes, paralysis ticks (*Ixodes holocyclus*), and cane toads (*Rhinella marina*) can result in SUD of companion animals. However, there are no publications which substantiate the impact of envenomations at a population level in companion animals with respect to postmortem investigation.

The aim of this study was to investigate the causes of SUD in companion animals in Australia, collating retrospective data from multiple university pathology services. This study examines SUD in the Australian context, providing useful data for Australian veterinarians and veterinary pathologists, and expanding the body of knowledge regarding SUD in cats and dogs.

## 2. Materials and Methods

### 2.1. Case Definitions

Based on the previous literature, sudden and unexpected death (SUD) was defined as death that occurred immediately or within 12 h of onset of acute clinical signs. This includes cases in which animals were found dead after being unobserved for up to 12 h and appeared normal before being unobserved.

Records were excluded if there was incorrect or missing information, history of illness in the lead-up to death, or no clinical history provided to indicate SUD had occurred. Animals under six months old were excluded to eliminate the neonatal death subpopulation and focus on adult SUD.

The cause of death was obtained by reviewing the postmortem report. Causes of death were grouped into the following major categories: cardiovascular, neoplasia, trauma, gastrointestinal, infection, iatrogenic, respiratory failure, toxic, endocrine, urinary, envenomation, and unknown. A combination of organ system and aetiology was used for categorization to allow for more clinically applicable results and meaningful data analysis. Cause of death was designated ‘unknown’ if a definitive cause of death could not be established following gross and histologic examination. If a cause of death fit more than one category, it was classified based on the most severe underlying disease process that led to death.

### 2.2. Data Collection

Canine and feline cases of SUD were obtained from four veterinary schools (referred to as ‘institutes’) in Australia, located at the University of Queensland (Queensland; QLD, Australia), the University of Melbourne (Victoria; VIC, Australia), Charles Sturt University (New South Wales; NSW, Australia), and the University of Adelaide (South Australia; SA, Australia). The study period was defined by the availability of digital reports for each institute. The University of Queensland Veterinary Laboratory Services database contained records from January 1994, and the University of Melbourne Veterinary Anatomic Pathology held digital records from January 1996. The University of Adelaide Veterinary Diagnostic Laboratory and Charles Sturt University Veterinary Diagnostic Laboratory held digital records from August and December 2012, respectively, due to the relatively recent establishment of these schools and their pathology services. The study period extended to June 2021 for all institutions.

Relevant post-mortem reports were extracted from databases and digital case files using the keywords and search phrases ‘sudden death’, ‘found dead’, ‘found deceased’, ‘dead on arrival’, ‘DOA’, and ‘died suddenly’. Results were restricted to dogs and cats. Records were obtained in this method from each school by the author on site, and all of the reports were manually reviewed by one author (M.K.B.) to determine suitability for inclusion. For the University of Queensland reports, data were exported directly into a Microsoft^®^ Excel^®^ spreadsheet, which was manually cleaned. For the other three laboratories, reports were received in PDF format, and reports which met the inclusion criteria were entered manually into the spreadsheet. Information displayed included a reference code, submission date, post code, species, breed, sex, neuter status, age, and cause of death ascertained from the post-mortem report.

### 2.3. Data Analysis

Cats and dogs were categorized into breed groups as per the Australian National Kennel Council (ANKC) and The Governing Council of the Cat Fancy. Animals were also categorized into age groups of four years old and under, five to nine years old, and over nine years old, as well as an unknown category for those without an age recorded. Submission dates were classified into Australian calendar seasons for temporal analysis: summer (December, January, and February), autumn (March, April, and May), winter (June, July, and August), and spring (September, October, and November).

Data analysis was conducted using the software Stata/SE 16.0 (StataCorp, College Station, TX, USA) and Microsoft Excel^®^. The frequency of SUD cases for each demographic variable (sex, neuter status, age group, season, and institute/location of the data source) was compared between species (dogs, cats) using the Fisher’s exact test.

Frequency of SUD was determined as the proportion of SUD in the total necropsy accessions of dogs and cats over the study period for each institute. The proportion of causes of SUD data were presented in bar charts.

The three most frequent SUD categories were charted against month of death, sex, neuter status, and age group. For month of death and institute, analysis was performed on the combined canine and feline study population. For the latter three variables, analysis was performed separately on each species. The Fisher’s exact test was used to determine if differences between the variables and the most frequent causes of SUD were significant.

The association of animal factors (sex, neuter status, age group, and breed group), season, and institute with the occurrence of SUD across the four most frequent SUD categories was explored using multinomial logistic regression. This analysis was performed separately for dogs and cats. Multinomial logistic regression analysis was performed using the Stata -*mlogit-* command and coefficients were presented as relative risk ratios (RRR), with the ‘cardiovascular’ category used as the base outcome. A Wald test was used to determine significance for the categorical variables. A forward and backward stepwise selection process was utilised to build the model, and variables that contributed significantly to the multivariate model (*p*-value < 0.05) were retained. Goodness-of-fit tests were performed using the Stata command -*mlogitgof*- [23], and predicted probabilities with 95% confidence intervals for each outcome were calculated using the -*margins*- and -*marginsplot*- commands.

## 3. Results

### 3.1. Study Population

A total of 575 records were extracted over the study period of January 1994 to June 2021, consisting of 339 records from the University of Queensland (QLD), 202 records from the University of Melbourne (VIC), 21 records from Charles Sturt University (NSW), and 13 records from the University of Adelaide (SA). The frequency of SUD in canine and feline necropsy accessions for each institute can be found in Table 1. For statistical analysis, records with incomplete information regarding sex and breed were excluded, leaving 558 records.

### 3.2. Signalment and Temporospatial Data

The records of SUD were composed of 424 (76%) dogs and 134 (24%) cats. Within the cat population, 33.6% (*n* = 45) were females and 66.4% (*n* = 89) were males, and the dog population contained 44.1% (*n* = 187) females and 55.9% (*n* = 237) males. Overall, 59.3% (*n* = 331) of the study population was neutered, including 84.3% (*n* = 113) of the cats and 51.4% (*n* = 218) of the dogs. There was a significant difference in sex (*p* = 0.035) and neuter status (*p* < 0.001) between dogs and cats that died from SUD.

In dogs, age ranged from 6 months to 16 years, with an average age of 5.17 years. Cats ranged in age from 6 months to 17 years, with an average age of 5.5 years. Overall, 46.2% (*n* = 258) of the study population was four years old or younger, 29.4% (*n* = 164) was between five and nine years old, and 16.3% (*n* = 91) was older than nine years. All of the breed groups of both cats and dogs were represented, and the count data for each group is outlined in Table 2. Working (*n* = 90, 21.2%), terrier (*n* = 87, 20.5%), and utility (*n* = 85, 20.0%) breeds were the most common dogs, and domestics (*n* = 87, 64.9%) were the most common cats.

Cases of SUD were submitted in every season of the year, with a range of 23% to 26% per season in the total study population (Table 2). There was a significant difference in the number of submissions per season between cats and dogs (*p* = 0.005). Cats had an increased frequency of SUD in winter (35%, *n* = 47) compared to dogs (24.3%, *n* = 103), and a decreased frequency in summer (15.7%, *n* = 21) compared to dogs (29.0%, *n* = 123).

### 3.3. Causes of Sudden Unexpected Death

**Figure 1:**
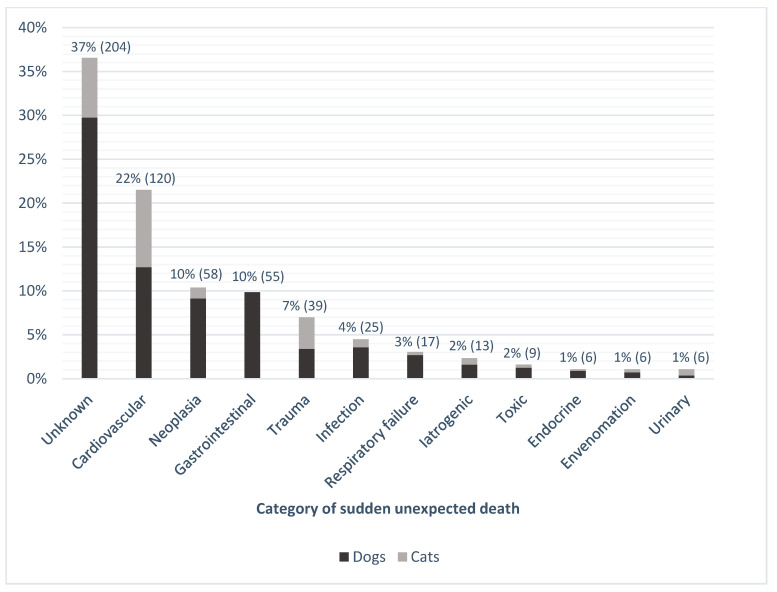
Percentage (number) of cases for the 12 categories of sudden unexpected death occurring in cats and dogs between 1994 and 2021 in four veterinary schools in Australia (*n* = 558).

There were 97 different diagnoses associated with SUD in the study population (Supplementary Materials Table S1). The most common category included records for which a cause of death was not definitively identified (‘unknown’), accounting for 37% (*n* = 204) of SUD cases (Figure 1). Where a cause was identified, cardiovascular disease was the most common cause of SUD in both cats and dogs (*n* = 120, 22%), which included entities such as hypertrophic and dilated cardiomyopathies, endocardiosis, and myocarditis (Supplementary Materials Table S1). The category with the next highest proportion was neoplasia (*n* = 58, 10%), followed by gastrointestinal disease (*n* = 55, 10%), and trauma (*n* = 39, 7%). Types of neoplasia resulting in sudden death included hemangiosarcoma (*n* = 35, 60%), lymphoma (*n* = 6, 10%), leukemia (*n* = 3, 5%), pheochromocytoma (*n* = 3, 5%), and pulmonary adenocarcinoma (*n* = 2, 3%). Only dogs had gastrointestinal disease as a cause of SUD, with 65% of the gastrointestinal deaths due to gastric dilatation and volvulus (GDV; *n* = 36). Trauma as a cause of SUD had a higher frequency in the feline population (*n* = 20, 15%) than the canine population (*n* = 19, 5%).

**Figure 2:**
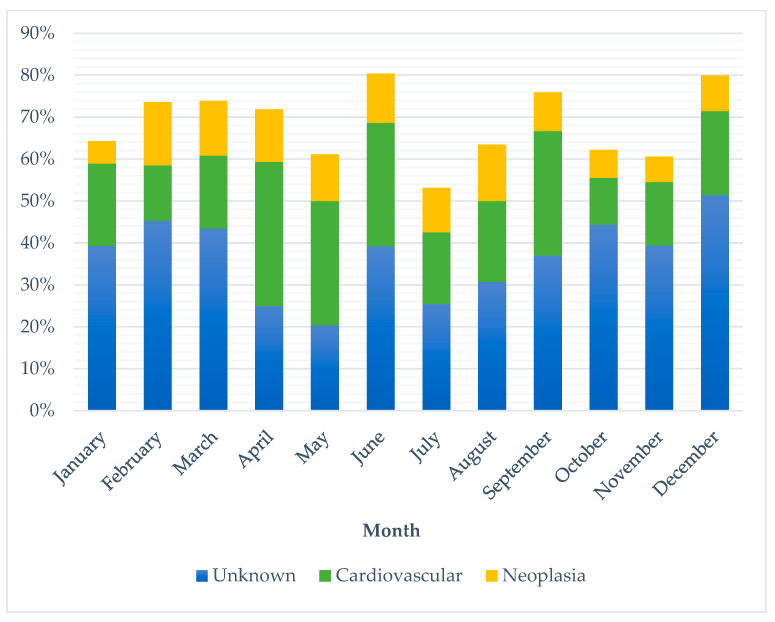
Percentage of occurrence of the three most common categories of sudden unexpected death in cats and dogs per month between 1994 and 2021 across four veterinary schools in Australia (*n* = 381).

**Figure 3:**
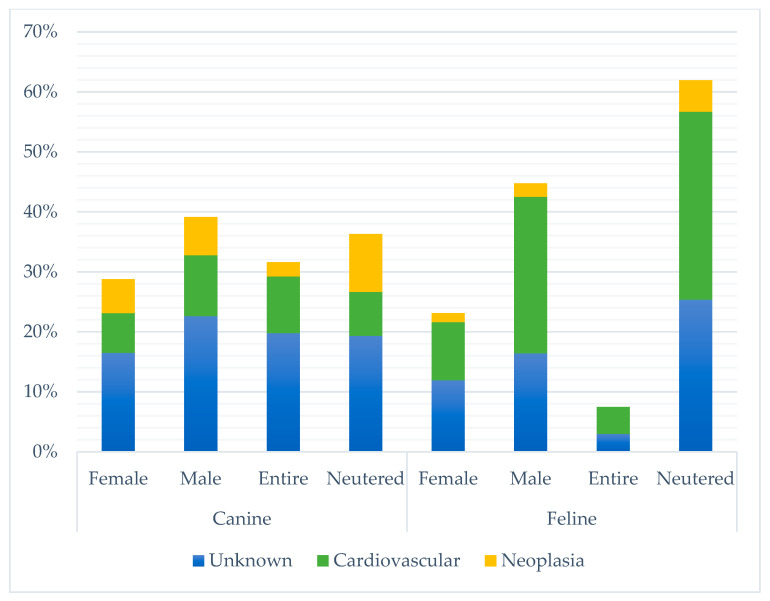
Percentage of occurrence of the three most common categories of sudden unexpected death by sex (canine *p*-value 0.713; feline *p*-value 0.215) and neuter status (canine *p*-value <0.001; feline *p*-value 1.000), stratified by species, between 1994 and 2021 across four veterinary schools in Australia (*n* = 381).

**Figure 4:**
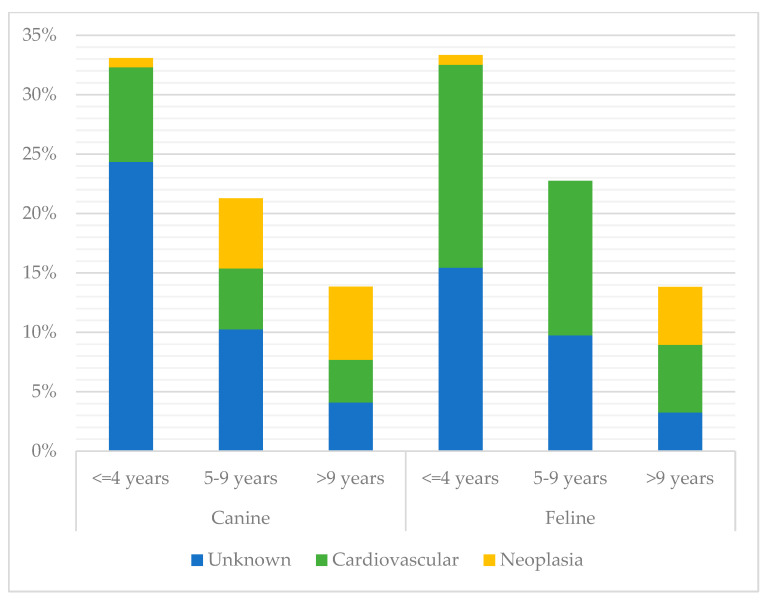
Percentage of occurrence of the three most common categories of sudden unexpected death by age group, stratified by species, between 1994 and 2021 across four veterinary schools in Australia (canine *p*-value <0.001; feline *p*-value 0.003; *n* = 352). Cases with unknown age were excluded.

Unknown SUD represented the largest proportion of deaths in every month except April and May, where cardiovascular disease had a higher frequency (see Figure 2). There was no significant difference between the largest three categories of SUD and month of death (*p*-value 0.315) or institute (*p*-value 0.823). Charles Sturt had a lower frequency of unknown SUD (19%) than other locations (34–39%). Frequencies of the three largest SUD categories also varied across sex and neuter status between cats and dogs (Figure 3). There was a significant difference between the top three SUD categories and age group in both cats and dogs (canine *p*-value <0.001; feline *p*-value 0.003), with frequency of unknown decreasing and neoplasia increasing as age increased (see Figure 4).

Within the unknown category, 65.2% (*n* = 133) of cases had a suspected or speculated cause of death based on the history and post-mortem findings (Table 3). Most commonly, cardiovascular SUD was suspected (*n* = 66, 32.4%), followed by a coagulopathy of unknown origin (*n* = 19, 9.3%), a toxin (*n* = 15, 7.4%), and envenomation (*n* = 13, 6.4%). In 34.8% (*n* = 71) of unknown SUD cases, there was no speculated cause. Marked autolysis was present in 15 of these cases, which hindered identification of pathologic lesions. A total of 3 cases had no lesions at all, and the remaining 53 cases had a range of non-specific lesions of varying severity, including congestion of various organs (*n* = 22); petechial to ecchymotic hemorrhage of various organs such as lung, pancreas and thymus (*n* = 24); and pulmonary oedema (*n* = 15).

### 3.4. Canine Multinomial Logistic Model

Age group was the only significant risk factor associated with the causes of death in dogs (Wald test *p*-value <0.001; Supplementary Materials Table S2 and Supplementary Materials Figure S1). When compared to the base outcome (cardiovascular disease), the RRR for neoplasia as the cause of SUD increased substantially as age increased. Conversely, the RRR for an unknown SUD compared to cardiovascular decreased with age. Those in the middle age group (5 to 9 years) had an increased RRR of gastrointestinal disease compared to cardiovascular disease. A goodness-of-fit statistic was unable to be generated due to the small sample size.

## 4. Discussion

This is the first multicentric study of sudden and unexpected death (SUD) in cats and dogs in Australia, spanning four states across varied climates and environments.

The frequency of SUD was similar between universities, with Adelaide (SA) recording the lowest frequency (2.14%) and Charles Sturt (NSW) recording the highest (6.46%). These two institutes had the smallest number of records contributing to the current study due to the recent establishment of their veterinary schools. Charles Sturt is the only institute included in this study which is not situated in a major city near the coast; it is located inland in NSW. It is possible that the rural location is a factor contributing to the slightly higher frequency of SUD. The bulk of the records originated from Queensland and Melbourne universities, which had very similar frequencies of SUD (5.48% and 4.54%, respectively). This consistency across four different states in Australia is notable and lends evidence that geographic location within the country has minimal impact on the causes of SUD in cats and dogs.

Multiple statistically significant differences were identified in the study population between cats and dogs. Overall, males accounted for a slightly larger proportion of SUD than females, and approximately two-thirds of cats were males (66.4%), which is higher than reported in the previous literature [12]. Potentially, male cats may be more predisposed to the common causes of SUD than females. In the current study, male cats had a much larger frequency of cardiovascular disease than females (Figure 3), which is consistent with a previous SUD study [13]. There is a known predisposition of males for hypertrophic cardiomyopathy [24], which was the most frequent cardiovascular disease causing SUD in this current study as well as in previous studies [12,13]. A significantly larger proportion of cats with SUD were neutered compared to dogs, which differed from the Canadian SUD studies in which the entire animal population composed of both cats and dogs outnumbered neutered [11,12]. This may reflect a change in frequency of desexing procedures over the decades. The higher proportion of neutered cats in the current study mirrors the general population of Australian cats under veterinary care [25,26]. Dogs had a more even distribution between neutered and entire in the current work, and the proportion of neutered dogs was lower than the reported desexing rate of dogs in Australia (81%) [27].

The average age and distribution across age groups were congruent in cats and dogs with SUD. Young animals were more frequently affected by SUD, with 46.2% of the study population aged four years old or younger, which is consistent with the Canadian literature [11,12]. This may be a true increased frequency, or could be influenced by submission bias because death at a younger age is more unexpected, and therefore is more likely to be investigated with a post-mortem examination. SUD occurred in every month of the year, and when grouped by season, there was a significant difference between cats and dogs, which differs from previous studies [11,12]. Any reasons why significantly more cats died in winter and more dogs died in summer in this current study are uncertain, as the etiologies that could be seasonal, most notably snake envenomations in the Australian context, would affect both cats and dogs. This discrepancy may be multifactorial, and could involve differences in movement restrictions or coping mechanisms for extreme ambient temperatures.

Numerous previously reported and novel etiologies of SUD were identified in this study. The frequencies of some conditions differed from the previous literature. Olsen and Allen (2001) reported the most frequent cause of SUD in cats was trauma, followed by cardiac disease [12]. These positions were reversed in the current work, with cardiovascular disease more frequent than trauma. The range of cardiac pathology observed in the current study is similar to that reported in the UK [13]. In Canadian dogs, Olsen and Allen (2000) reported heart disease and toxicity as the two most common causes of SUD, followed by gastrointestinal disease and trauma [11]. The current Australian data also found cardiovascular disease to be the most common cause; however, toxicities accounted for only a very small number of SUD cases in this current work. Neoplasia also had a higher frequency in the current study than was previously reported, although the same type of neoplasia (hemangiosarcoma) was still most frequent in both. In addition, the current study expanded the range of neoplasia that can be associated with SUD to include lymphoma, leukemia, and pheochromocytoma. In both cats and dogs, snake envenomation and tick paralysis were causes of SUD in the current work that had not been previously reported.

Unknown cause of SUD was the most frequent of all categories in both cats and dogs, accounting for more than one third of deaths in the whole study population. This is higher than described in the Canadian, UK, and Italian papers, where 12.6% to 14.5% were reported [10,11,12,13]; and more similar to the SUD frequency reported in horses [17,19]. This difference between the current work and previous publications reinforces the necessity for this Australian-based study. A high rate of unknown SUD in Australia compared to other countries is likely multifactorial. Firstly, there is an abundance of venomous or poisonous flora and fauna native to Australia. Exposure to a variety of snakes and spiders, cane toads, paralysis ticks, toxic vegetation, fungi, and cyanobacteria can result in death with minimal lesions [28]. Additionally, there is a lack of toxin-specific assays available to diagnose these agents. In the Canadian paper, toxicities were limited to two agents: strychnine and carbon monoxide [11]. In Australia, there are numerous toxic causes and limited testing availability, thus, intoxication often cannot be confirmed. The high ambient temperature in Australia is also likely to be a factor impacting diagnostic success. High ambient temperatures speed autolysis, which impedes post-mortem examination by obscuring pathologic lesions. Autolysis was a cited factor preventing a definitive diagnosis in 15 of the unknown SUD reports in this study. Delayed refrigeration and transport to the necropsy facility provides time for autolysis to occur, which is expedited by warm climate. Cadavers may be frozen by clinicians to prevent autolysis if there is a lack of a refrigeration option, which causes further artefacts. Financial factors also play a role in the pro bono necropsies performed for veterinary student teaching purposes. In these cases, financial constraints preclude the use of further testing modalities such as microbiology or toxicology, and therefore, these cases may be more likely to remain unresolved.

Identification of non-specific or agonal lesions was common in the unknown SUD cases, which reported lesions such as pulmonary oedema, congestion, and thymic or pancreatic hemorrhages. These lesions may be considered agonal if there is no cause identified [28]. Additionally, pink fluid may accumulate in alveoli post-mortem, which can be spuriously interpreted as pulmonary oedema [29]. Etiologies that act at a molecular level to result in death do not have lesions observable by light microscopy, either due to the time required for morphologic change to manifest in diseased tissue or because the mechanism of action does not cause cell injury (e.g., botulism). Changes observed by histopathology take time to develop following initial injury. In myocardial infarcts, microscopic lesions take 6 to 12 h to develop [30], and in a mouse model of acetaminophen toxicity, hepatocytes showed damage at 5 h post dosage at the earliest [31]. This suggests that when death occurs within a few hours of insult, it is possible for no lesions to be visualized microscopically. Additionally, the methodology of examination and sampling the heart at necropsy varies, and standardization of the process may allow for increased recognition of cardiac lesions.

One role of post-mortem examination is to diagnose a cause of death. The high proportion of animals where post-mortem examination failed to achieve a definitive diagnosis in this study was unexpected. This may reflect the Australian context, including preservation artefacts, the higher proportion and impact of toxicologic etiologies, and the lack of specific cost-effective assays for many toxicants. It is considered unlikely that the skill of the veterinary pathologists in Australia is a factor in the high unknown rate, as pathologists with different levels of experience performing necropsies are global.

Multinomial logistic regression was used to model potential risk factors for the four most frequent categories of sudden death, with the intent to conceive a diagnostic algorithm to assist veterinary pathologists with investigation of SUD cases. The feline subset of the population was too small to obtain meaningful results with modelling. In dogs, age group was the only variable associated with the outcomes and thus, was the only variable included in the model. The model determined that the relative risk of dying suddenly from neoplasia rather than cardiovascular disease (the base outcome) increased substantially with increased age. Further, the relative risk of dying due to unknown SUD instead of cardiovascular disease decreased with age. This is mirrored by the relative proportions of these SUD categories in each age group (Figure 4). Neoplasia was significantly more common as age increased, and unknown SUD was significantly less common as age increased. The proportion of cardiovascular disease did not vary greatly between age groups.

A few of the causes of SUD identified at necropsy (see Supplementary Materials Table S1) are not typically associated with rapid death and usually have premonitory signs, such as portosystemic shunt and hyperthyroid. In humans, SUD may occasionally be caused by a seemingly chronic disease such as hepatic cirrhosis [8]. In the current study, it is possible that some of these chronic disease diagnoses were incidental and did not result in death, or they may have presented as SUD because clinical signs went unrecognized. This is one of the drawbacks of retrospective data collection, and reflects the complicated nature of defining ‘cause of death’ and ‘sudden death’.

This study was limited by the small population size, particularly in cats. However, this was compensated for by the broad geographic distribution of the datasets and the multicentre design. The retrospective nature of this study allowed for a temporal range of dataset accrural, but had inherent constraints, such as variable consistency in reports, missing information, variation in what tissues were examined by histopathology, and what ancillary tests were performed. The potential to expand this study exists, either by recruiting other veterinary schools in Australia and overseas to supplement this retrospective study, or by collaboration to conduct a prospective study of SUD. A prospective study could implement a standardized approach to necropsy and ancillary testing which may increase the rate of definitive diagnosis.

## 5. Conclusions

To the authors knowledge, this is the first multicentric study detailing the epidemiology and pathology of SUD in cats and dogs in Australia. There was a statistically significant difference in the sex, neuter status, and season of death between cats and dogs presenting for necropsy for SUD. The most frequent category of SUD was the unknown group, followed by cardiovascular disease, neoplasia, and gastrointestinal disease. On multinomial logistic regression modelling for dogs, there was an increased relative risk for neoplasia compared to cardiovascular disease with increased age, and a decreased relative risk of unknown SUD. This study provides insight into SUD in cats and dogs in Australia.

## Figures and Tables

**Table 1:** Frequency of sudden unexpected death for each institute included in the study (calculated prior to incomplete record exclusion).

Institute	Study Period	Total Necropsy Accessions (Combined Dogs and Cats)	SUD Count	Frequency of SUD by Necropsy Submissions (Combined Dogs and Cats)
Queensland	Jan 1994–Jun 2021	6184	339	5.48%
Melbourne	Jan 1996–Jun 2021	4454	202	4.54%
Adelaide	Aug 2012–Jun 2021	607	13	2.14%
Charles Sturt	Dec 2012–Jun 2021	325	21	6.46%

**Table 2:** Demographics (number of cases, percentage of cases) of sudden unexpected death cases occurring in cats and dogs between 1994 and 2021 across four veterinary schools in Australia. P values indicate the comparison of demographics between cases in cats versus dogs.

Variable	Overall	Cats (*n* = 134)	Dogs (*n* = 424)	*p*-Value
Sex				
Female	232 (41.6)	45 (33.6)	187 (44.1)	0.035
Male	326 (58.4)	89 (66.4)	237 (55.9)	
Neuter status				
Entire	227 (40.7)	21 (15.7)	206 (48.6)	<0.001
Neutered	331 (59.3)	113 (84.3)	218 (51.4)	
Age group				
<=4 years	258 (46.2)	64 (47.8)	194 (45.7)	0.635
5–9 years	164 (29.4)	34 (25.4)	130 (30.7)	
>9 years	91 (16.3)	25 (18.6)	66 (15.6)	
Unknown	45 (8.1)	11 (8.2)	34 (8.0)	
Breed group				
Gundog	-	-	39 (9.2)	N/A
Hound	-	-	54 (12.7)	
Non-sporting	-	-	37 (8.7)	
Terrier	-	-	87 (20.5)	
Toy	-	-	32 (7.5)	
Utility	-	-	85 (20.0)	
Working dog	-	-	90 (21.2)	
British	-	8 (6.0)	-	
Burmese	-	6 (4.5)	-	
Domestic	-	87 (64.9)	-	
Foreign	-	10 (7.5)	-	
Persian	-	10 (7.5)	-	
Semi-longhair	-	12 (9.0)	-	
Siamese/oriental	-	1 (0.7)	-	
Season				
Summer	144 (25.8)	21 (15.7)	123 (29.0)	0.005
Autumn	132 (23.7)	30 (22.4)	102 (24.1)	
Winter	150 (26.8)	47 (35.0)	103 (24.3)	
Spring	132 (23.7)	36 (26.9)	96 (22.6)	
Institute (Location)				
UQ (QLD)	329 (59.0)	80 (59.7)	249 (58.7)	0.996
UM (VIC)	195 (35.0)	46 (34.3)	149 (35.1)	
CSU (NSW)	21 (3.7)	5 (3.7)	16 (3.8)	
UA (SA)	13 (2.3)	3 (2.3)	10 (2.4)	

**Table 3:** List of the suspected causes of unknown sudden unexpected death.

Suspected Causes of Unknown SUD	Count (*n*,%)
No suspected cause	71 (34.8)
Cardiovascular	66 (32.4)
Coagulopathy	19 (9.3)
Toxic	15 (7.4)
Envenomation	13 (6.4)
Iatrogenic	7 (3.4)
Respiratory failure	7 (3.4)
Endocrine	3 (1.5)
Gastrointestinal	2 (1.0)
Infection	1 (0.5)
Total	204

## Data Availability

The data presented in this study are available on request from the corresponding author.

## Supplementary Materials

The following supporting information can be downloaded at: https://www.mdpi.com/article/10.3390/vetsci10090582/s1, Table S1: Complete list of the diagnoses associated with sudden unexpected death in cats and dogs in this study; Table S2: Final multinomial logistic regression model of the risk factor significantly associated with the outcomes (the highest frequency causes of sudden unexpected death) in dogs; Figure S1: Predicted probabilities (with 95% confidence intervals) of the risk factor age group associated with the outcomes (a) cardiovascular, (b) gastrointestinal, (c) neoplasia, and (d) unknown as the cause of sudden unexpected death.
