# Supplementary material for: A Multicentre Epidemiologic Study of Sudden and Unexpected Death in Adult Cats and Dogs in Australia
Source: Vet Sci. 2023 Sep 20;10(9):582. doi: 10.3390/vetsci10090582 (PMC10535394; doi:10.3390/vetsci10090582)
Supplement: Supplementary file 1 [file vetsci-10-00582-s001.zip › vetsci-2523326-supplementary.pdf]

Table S1. Complete list of the causes of SUD in cats and dogs observed in this study.

Causes of SUD		Cats (n=134)	Dogs (n=424)
**Category**	**Cause**		
Cardiovascular		***48***	***71***
	Arrhythmogenic right ventricular cardiomyopathy	0	2
	Atrial rupture	0	1
	Cardiomyopathy, other	7	16
	Cardiovascular collapse	0	1
	Chordae tendinae rupture	0	2
	Congestive heart failure	0	3
	Dehydration	0	5
	Dilated cardiomyopathy	0	7
	Endocardial fibroelastosis	1	0
	Endocardiosis	0	9
	Endocarditis	0	1
	Hemoabdomen	0	2
	Hemopericardium	1	3
	Hemothorax	0	1
	Heartworm	0	1
	Heat stroke	0	3
	Hypertrophic cardiomyopathy	28	0
	Myocarditis	4	1
	Pericardial effusion	0	1
	Peritoneopericardial hernia	1	1
	Portosystemic shunt	0	1
	Pulmonary hypertension	1	0
	Pulmonary thromboembolism	3	0
	Shock	0	1
	Splenic rupture	0	3
	Subaortic stenosis	0	3
	Thrombosis	0	1
	Valvular dysplasia	2	1
	Vascular anomaly	0	1
Endocrine		***2***	***5***
	Adrenal necrosis	0	1
	Hyperthyroid	2	0
	Hypoadrenocorticism	0	4
Envenomation		***2***	***4***
	Snake envenomation	1	3
	Tick paralysis	1	1
Gastrointestinal		***0***	***55***
	Acute pancreatic necrosis	0	1
	Gastric dilation	0	3
	Gastric perforation	0	1
	GDV	0	36
	Hemorrhagic enteritis	0	1
	Hemorrhagic gastroenteritis	0	1
	Hepatic encephalopathy	0	1
	Hookworm	0	1
	Intestinal foreign body	0	3
	Intestinal perforation	0	1

	Mesenteric volvulus	0	5
	Necrotizing enteritis	0	1
Iatrogenic		***4***	***9***
	Anesthetic-associated death	4	6
	Post-operative hemoabdomen	0	3
Infection		***5***	***20***
	Abscess and hemothorax	0	1
	Actinomycotic pleuritis	0	1
	Aspiration pneumonia	1	2
	Cryptococcal meningitis	1	0
	Meningoencephalitis	0	1
	Mycotic aneurysm	0	1
	Necrotizing metritis	0	1
	Parvovirus	0	1
	Peritonitis	0	1
	Phaeohyphomycosis	0	1
	Pneumonia	2	4
	Pyelonephritis & septicemia	0	1
	Pyometra	0	3
	Septic shock	0	2
	Tyzzer's disease	1	0
Neoplasia		***7***	***51***
	Adrenal neoplasia	0	1
	Chondrosarcoma	0	1
	Hemangiosarcoma	1	34
	Hepatocellular carcinoma	0	1
	Histiocytic sarcoma	0	1
	Leukemia	0	3
	Lymphoma	3	3
	Neurofibrosarcoma	0	1
	Oligodendroglioma	0	1
	Pancreatic adenocarcinoma	0	1
	Pheochromocytoma	0	3
	Pulmonary adenocarcinoma	2	0
	Splenic mast cell tumor	1	0
	Thymoma	0	1
Respiratory failure		***2***	***15***
	Asphyxiation	1	10
	BOAS	0	1
	Feline asthma	1	0
	Laryngeal oedema	0	2
	Pulmonary fibrosis	0	1
	Spontaneous pneumothorax	0	1
Toxic		***2***	***7***
	1080 toxicity	0	2
	Hepatotoxicity	1	0
	Metaldehyde toxicity	0	2
	Nephrotoxicosis	1	0
	Organochloride toxicity	0	1
	Rodenticide toxicity	0	1
	Toad toxicity	0	1
Trauma		***20***	***19***

Urinary		***4***	***2***
	Acute renal tubular necrosis	1	0
	End-stage chronic renal failure	0	1
	FLUTD	2	0
	Systemic amyloidosis	1	0
	Urinary bladder rupture	0	1
Unknown		***38***	***166***

Table S2. Multinomial logistic regression model of the risk factor significantly associated with the outcomes (the highest frequency causes of SUD) in dogs. The base outcome is cardiovascular disease.

	Gastrointestinal			Neoplasia			Unknown		
**Risk factor**	**RRR (CI 95%)**	**P-value**	**Wald test**	**RRR (CI 95%)**	**P-value**	**Wald test**	**RRR (CI 95%)**	**P-value**	**Wald test**
Age									
4 years and under	1 (reference)		0.056	1 (reference)		<0.001	1 (reference)		0.054
5 to 9 years	2.62 (1.13,6.03)	0.024		11.88 (3.15,44.84)	<0.001		0.65 (0.33,1.28)	0.214	
Over 9 years	1.11 (0.38,3.19)	0.850		17.71 (4.56,68.75)	<0.001		0.37 (0.16,0.85)	0.019	

RRR = Relative risk ratio

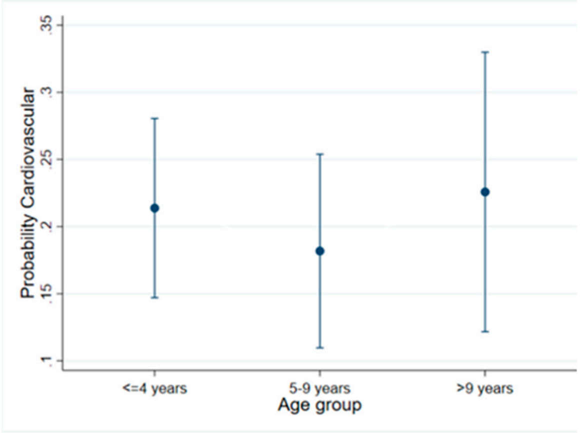

(a)

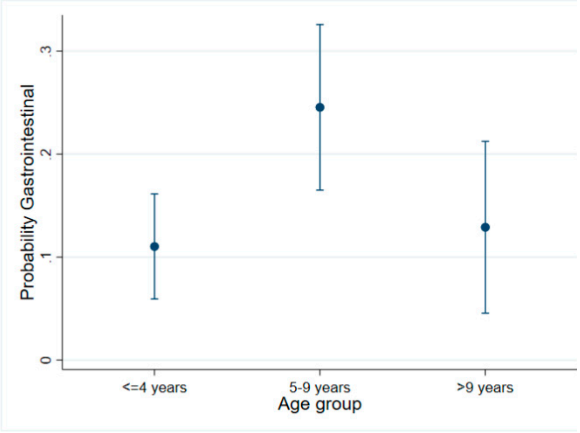

(b)

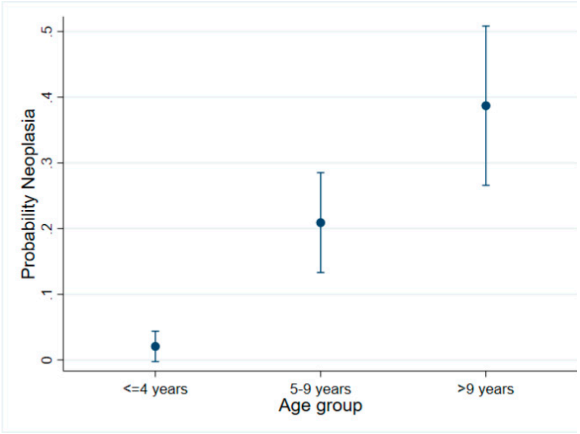

(c)

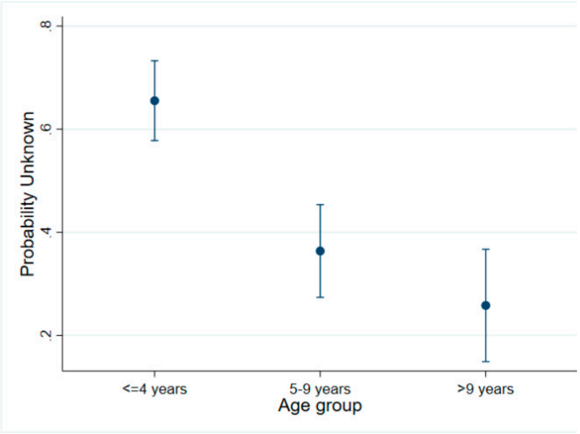

(d)

Figure S1. Predicted probabilities (with 95% confidence intervals) of the risk factor age group associated with the outcomes (a) cardiovascular, (b) gastrointestinal, (c) neoplasia, and (d) unknown as the cause of SUD.
